# METTL3/IGF2BP2 axis affects the progression of colorectal cancer by regulating m6A modification of STAG3

**DOI:** 10.1038/s41598-023-44379-x

**Published:** 2023-10-12

**Authors:** Jianmei Yi, Feng Peng, Jingli Zhao, Xiaosong Gong

**Affiliations:** 1 The Department of General Surgery 2, Zhuzhou Central Hospital (Zhuzhou Hospital Affiliated to Xiangya School of Medicine, Central South University), 116 Changjiang South Road, Tianyuan District, Zhuzhou, 412007 Hunan China; 2 The Department of Operating Room, Zhuzhou Central Hospital (Zhuzhou Hospital Affiliated to Xiangya School of Medicine, Central South University), Zhuzhou, 412007 China

**Keywords:** Colorectal cancer, Colorectal cancer

## Abstract

Colorectal cancer (CRC) is among the commonest malignant tumors of humans. Existing evidence has linked the poor prognosis of CRC with high expression of stromal antigen 3 (STAG3), but, the exact biological effect of STAG3 in CRC is still unclear. The aim of this research is to reveal the biological function and molecular mechanism of STAG3 in CRC. To investigate the differential expression of STAG3 in CRC tissues and cell lines compared to normal colon tissues and cell lines, Western blot (WB) and quantitative real-time PCR (qRT-PCR) techniques were utilized. STAG3 *N*6-methyladenosine (m6A) modification level were identified using m6A RNA immunoprecipitation (MeRIP). Additionally, the functional roles of methyltransferase-like protein 3 (METTL3) and insulin-like growth factor 2 mRNA binding protein 2 (IGF2BP2) in CRC were explored by manipulating their levels via knockdown or overexpression. Cell proliferation was evaluated through Cell Counting Kit 8 (CCK-8) and clone formation experiments, while cell migration was assessed through wound healing experiments. Furthermore, cell apoptosis was detected using flow cytometry, and the protein expressions associated with proliferation and apoptosis were detected using WB. To identify the specific binding of target genes, RIP and pull-down assays were employed. Finally, the biological function of STAG3 in vivo was investigated through a xenotransplantation mouse tumor model. In CRC tissues and cell lines, STAG3 was up-regulated and accompanied by m6A methylation. Additionally, the expression of METTL3 was found to be upregulated in CRC tissues. Knocking down METTL3 resulted in a decrease in both the m6A level and protein expression of STAG3, inhibited cell proliferation and migration while promoting apoptosis, which were restored through STAG3 overexpression. Furthermore, online prediction indicated the interaction between STAG3 mRNA and IGF2BP2 protein, which was further verified by RIP experiments. IGF2BP2 downregulation led to decreased STAG3 protein expression, cell proliferation, and migration, but increased apoptosis. However, these impacts were reversed by STAG3 overexpression. Finally, subcutaneous tumor experiments conducted in nude mice also confirmed that METTL3 regulated CRC progression through STAG3 in vivo. The METTL3/IGF2BP2/STAG3 axis affects CRC progression in an m6A modification-dependent manner. This may guide targeted therapy in CRC patients.

## Introduction

Colorectal cancer (CRC) is the most prevalent gastrointestinal malignancy and the second largest cause of cancer-related mortality globally^1^. It is a significant public health issue because of rising morbidity and death, with high mortality mainly caused by tumor spread and recurrence^2,3^. The process of tumor metastasis is complex and dynamic, and it depends on several factors. Therefore, further research is urgently needed to characterize the potential molecular mechanisms involved in CRC metastasis and to develop new biomarkers and treatment methods.

Stromal antigen 3 (STAG3), the main subunit of the meiosis-specific adhesion protein complex, controls sister chromatid adhesion^4^. There is proof that a poor prognosis for CRC is linked with increased STAG3 expression^5^. Similarly, *N*6-methyladenosine (m6A), a methylation modification at the nitrogen-6 position of adenine base, is the most frequent mRNA modification. The m6A is crucial for regulating the splicing, translation, and degradation of mRNA^6–8^. The m6A enzyme system, which is mostly made up of methyltransferase (''writer''), demethylase (''eraser''), and binding protein (''reader''), exerts a function in the pathophysiology and development of cancer through m6A modification^9,10^. Studies have shown that m6A has a great importance in tumor spread of diverse cancer types^9,11,12^. However, whether STAG3 expression in CRC is regulated by m6A modification and its related enzymes remains to be studied.

Methyltransferase-like 3 (METTL3) is a major m6A methyltransferase. It has been shown to play a significant role in tumor progression through a number of downstream genes in hepatocellular carcinoma^13^, leukemia^14^, lung cancer^15^, and malignant glioma^16^. In addition, Li et al. revealed the promoting effect of METTL3 on CRC progression through the m6A-dependent mechanism^17^. Similarly, insulin-like growth factor 2 binding protein 2 (IGF2BP2) is a crucial m6A modification "reader". It contributes to the development of a multitude of malignant tumors by post-transcriptionally controlling the stability and translation of its main target mRNA^18,19^. Hence, IGF2BP2 is a crucial tumor-promoting factor in the development of CRC, according to studies^20,21^.

In this study, we aim to demonstrate that STAG3 is expressed differently in CRC versus normal tissues through clinical sample collection. Based on this, in vitro experiments were applied to analyze the effects of STAG3 on the proliferation, migration, and apoptosis of CRC cells. Also, in vivo experiments were carried out to further confirm these findings. In addition, we further explored the potential mechanism of STAG3 in CRC. The study also aims to provide new understanding in the development of potential treatment strategies for CRC.

## Materials and methods

### Clinical sample collection

A total of 30 cases of CRC tissue samples and 30 cases of adjacent normal tissue samples were collected from Zhuzhou Central Hospital. All patients did not receive radiotherapy or preoperative chemotherapy. All samples were taken with the patient's informed permission. Also, and all procedures were authorized by the Ethics Committee of Zhuzhou Central Hospital (No. ZZCHEC2020084-01).

### Cell culture and transfection

The human CRC cell lines HCT116 (AW-CCH026), HT29 (AW-CCH054), SW480 (AW-CCH107), LOVO (AW-CCH027), and SW620 (AW-CCH140), and normal colon cell line NCM460 (AW-CCH306) were purchased from Abiowell (China). HCT116 and HT29 cells were cultured in McCoy’s 5A medium (iCell-0011, iCell Bioscience Inc., China). SW480 cells were cultured in Leibovitz's L-15 medium (AW-MCO12). LOVO cells were cultured in F12K medium (AW-MCO04). SW620 cells were cultured in DEME medium (AW-MC002). NCM460 cells were cultured in RPMI-1640 medium (AW-M002). All medium were supplemented with 10% fetal bovine serum and 1% penicillin–streptomycin. The culturing temperature for all cell lines was set at 37 °C. Except for SW480 cells, which were cultured under no CO_2_ conditions, all other cells were cultured under conditions containing 5% CO_2_. sh-METTL3, oe-METTL3, oe-STAG3, sh-IGF2BP2, and oe-IGF2BP2 were transfected into HCT116 and SW620 cells in accordance with the manufacturer's instructions using Lipofectamine 2000 (11668019, Invitrogen, USA). oe-NC and sh-NC were used as the blank controls. The abbreviations oe, sh, and NC represent overexpression, shRNA, and negative control, respectively. The culture medium was changed after 6 h of transfection, and the related detection was performed after 48 h.

### Quantitative real-time PCR (qRT- PCR)

Utilizing the TRIzol reagent (15596026, Thermo Fisher Scientific, USA), total RNA was extracted from tissues and cells. Following a search for the target gene's sequence on NCBI, the primers were constructed using the primer 5 software. Using the mRNA reverse transcription kit (CW2569, CWBIO, China), the extracted total RNA served as a template to reverse the cDNA. An UltraSYBR Mixture (CW2601, CWBIO, China), cDNA, and primer qRT-PCR system were used in the qRT-PCR (QuantStudio1, Thermo Fisher Scientific, USA). As an internal reference, β-actin was used. The relative expression was estimated by 2^−ΔΔCt^ method. The sequence of the primers is displayed in Table 1.

**Table 1:** Primer sequences.

Gene	Primer sequences	Length (bp)
STAG3	FCAGCTCCACTCCCTACCTCA	157
	RGCTTCTCTACCCCAGAGGGA	
METTL3	FATCCCCAAGGCTTCAACCAG	119
	RGCGAGTGCCAGGAGATAGTC	
β-actin	FACCCTGAAGTACCCCATCGAG	224
	RAGCACAGCCTGGATAGCAAC	

### Western blot (WB)

To extract total protein from tissues and cells, RIPA buffer (P0013B, Beyotime, China) was adopted. The BCA protein quantification kit was utilized to calculate the total protein content. After electrophoretic separation, the protein supernatant was moved to nitrocellulose membrane. To block the membrane for 1.5 h at room temperature, 5% skimmed milk powder was used. The membrane was then incubated overnight at 4℃ with primary antibodies targeting STAG3, METTL3, METTL14, ALKBH5, FTO, PCNA, Ki67, Bcl-2, Bax, caspase3, and IGF2BP2, using β-actin as the internal reference. The membrane was washed thrice with PBST and incubated at room temperature for 1.5 h with secondary antibodies (HRP goat anti-mouse IgG and HRP goat anti-rabbit IgG). After incubation, PBST was washed three times. We used ECL chromogenic substrate (AWB0005, Abiowell, China) to visualize protein bands. For scanning and imaging, chemiluminescence imaging was used. Antibody information is shown in Table 2.

**Table 2:** Antibodies used in the study are shown.

Antibodies	Dilution	Origin	Mw (KDa)	Manufacturer
STAG3	0.4 μg/mL	Rabbit	139	ab185109, Abcam
METTL3	1: 1000	Rabbit	65–70	15073-1-AP, Proteintech
METTL14	1: 1000	Rabbit	55	orb28269, Biorbyt
ALKBH5	1: 5000	Rabbit	40–50	16837-1-AP, Proteintech
FTO	1: 1500	Rabbit	58	27226-1-AP, Proteintech
PCNA	1: 5000	Rabbit	36–38	10205-2-AP, Proteintech
Ki67	1: 1000	Rabbit	358	ab16667, Abcam
Bcl-2	1: 500	Rabbit	26	26593-1-AP, Proteintech
Bax	1: 5000	Rabbit	21	50599-2-Ig, Proteintech
caspase3	1: 1000	Rabbit	32–35, 17, 19	19677-1-AP, Proteintech
IGF2BP2	1: 8000	Rabbit	55–65	11601-1-AP, Proteintech
β-actin	1: 5000	Mouse	42	66009-1-Ig, Proteintech
HRP goat anti-mouse IgG	1: 5000	Mouse	–	SA00001-1, Proteintech
HRP goat anti-rabbit IgG	1: 6000	Rabbit	–	SA00001-2, Proteintech

### RNA immunoprecipitation (RIP) and m6A-RIP (Me-RIP) assays

RIP analysis using a RIP assay kit (Catalog No. 17-700, Millipore, USA), was conducted as directed by the manufacturer. In short, cells were treated with lysates and incubated overnight at 4℃ with magnetic beads coated with anti-Argonaute (ab32381, Abcam). Following that, proteinase K was added and the beads underwent six rounds of washing. The RNA from immunoprecipitates and inputs was extracted using the TRIzol-chloroform-isopropanol reagent. Using qRT-PCR, the precipitated RNA was examined. To assess the specificity of RNA–protein interactions, IgG was utilized after relative expression had been normalized to input. For Me-RIP, the m6A methylation rate of STAG3 was detected by using STAG3 extracted from cells with lysate as input. The antibodies used in this experiment were anti-m6A (ab286164, Abcam) and IGF2BP2 (11601-1-AP, Proteintech).

### Cell counting kit 8 (CCK-8) assay

Cells were digested and counted during the logarithmic growth phase. 1 × 10^4^ cells/well, in 100 μL each well, were planted on 96-well plates (0030730119, Eppendorf, Germany). Each group had three duplicate holes. The medium was changed after the cells attached to the well, and the appropriate intervention was carried out. After culture for the corresponding time, the medium was discarded. Each well should contain 10 μL of CCK-8 solution (NU679, DOJINDO, Japan). The Bio-Tek microplate reader (MB-530, Heales, China) was employed to measure the absorbance value at 450 nm, and the mean value was taken as the histogram.

### Clone formation assay

Cells were trypsinized. In a 6-well plate with 1 mL of culture medium that has been brought to room temperature, the cell suspension was inoculated at a density of 200 cells per well. Cells were cultured in a 5% CO_2_ incubator at 37 ℃ for 2–3 weeks, and the medium was changed appropriately. The culture is stopped when there are obvious clones in the culture plate. After discarding the culture medium, the PBS solution underwent two thorough washes. 1 mL 4% paraformaldehyde (N1012, NCM Biotech Co., Ltd, China) was introduced to each well and fixed for 15 min. The fixative was discarded, and 1 mL crystal violet staining solution (G1062, Solarbio, China) was added for 30 min at room temperature. The dye solution was rinsed slowly with running water, and the samples were photographed after drying naturally.

### Wound healing assay

A 6-well plate was taken, and a horizontal line was drawn evenly behind the plate. After trypsinization and counting, the cells were seeded in 6-well plates at a density of 5 × 10^5^ cells/well. After the cells filled the plate, scratches were made perpendicular to the previous horizontal line. Cell debris was removed with PBS. McCoy’s 5A medium with low serum was added and cultured at 37 ℃, 5% CO_2_. The scratch widths at 0, 24 and 48 h were observed and recorded using an inverted biological microscope (DSZ2000X, Cnmicro, China), and three fields of view were taken at each time point.

### Flow cytometry

Apoptosis was detected by flow cytometry. The first step in the exact gating strategy for the flow cytometric analysis is to confirm the location of negative signal using an unlabeled negative sample. Then, common single staining experiments, (such as APC and PI staining) are performed to determine the gate position. Finally, by determining the threshold and position of the apoptotic cells, accurate results of flow cytometric apoptosis analysis can be obtained. In short, Trypsin digestion was performed on each group's cells without the addition of EDTA. About 3.2 × 10^5^ cells were collected after the cells have been centrifuged twice at 2000 rpm for 5 min each time. With 500 μL of binding buffer, the cells were suspended. 5 μL of Annexin V-fluorescein isothiocyanate (KGA108, KeyGen, China) was added and blended with 5 μL of propidium iodide. Cells were incubated in dark at room temperature for 10 min. The cells were analyzed by flow cytometry (A00-1-11102, Beckman, USA) during 1 h.

### Immunohistochemistry (IHC)

After dewaxing and rehydration, the sections were immersed in 0.01 M citrate buffer (pH 6.0), and heated in a microwave to restore the antigen. After reacting at room temperature for 10 min, the sections were treated with PBS and 1% periodate. The sections were treated with the primary antibody PCNA (10205-2-AP, 1:500, Proteintech) overnight at 4 °C before being incubated with the secondary antibody for 30 min the following day. The sections were stained with DAB, washed with PBS, counterstained with hematoxylin for 5–10 min, washed with distilled water, and finally returned to blue with PBS. Gradient alcohol (60–100%) was used for dehydration, 5 min per stage. After removal, the sections were placed in xylene for 10 min. Finally, the neutral gum was applied to seal sections and the microscope was utilized to observe.

### Animal experiment

Male BALB/c nude mice (4 weeks, 12~15 g), were ordered from Hunan SJA Laboratory Animal Co., Ltd. oe-STAG3 and sh-METTL3 were transfected into HCT116 cells. Trypsin digestion and cell counting were performed on each set of cells after they had reached around 80% confluence. Nude mice were subcutaneously injected with 200 μL cells (2 × 10^6^) and randomly assigned to 5 groups (n = 6 in each group): control group (mice were injected with cells without transfection plasmid), oe-NC group (mice were injected with cells transfected with oe-NC), oe-STAG3 group (mice were injected with cells transfected with oe-STAG3), oe-STAG3 + sh-NC group (mice were injected with cells transfected with oe-STAG3 and sh-NC) and oe-STAG3 + sh-METTL3 group (mice were injected with cells transfected with oe-STAG3 and sh-METTL3). Following injection, the tumor volume was computed using the following equation: volume = 1/2 × (width^2^ × length). Tumor size was then evaluated every five days using a digital Vernier caliper. The mice were euthanized by intraperitoneal injection of excessive pentobarbital sodium (200 mg/kg) at the conclusion of the animal experiment^22^. The Institutional Animal Care and Use Committee, The Second Xiangya Hospital, Central South University (Approval No. 2020398), gave the approval for all animal experiments. All methods were carried out in accordance with ARRIVE guidelines. The above-mentioned experiments were conducted at least three times.

### Statistical analysis

The data was analysed using the software GraphPad Prism 8.0, all data findings were assessed. A one-way ANOVA was employed to compare various data sets. Statistical significance was set at P < 0.05.

### Ethics approval

The study was conducted according to the guidelines of the Declaration of Helsinki. The use of clinical samples was approved by the Ethics Committee of Zhuzhou Central Hospital (No. ZZCHEC2020084-01), and the animal research was approved by the Institutional Animal Care and Use Committee, The Second Xiangya Hospital, Central South University (No. 2020398). All methods were carried out in accordance with relevant guidelines and regulations.

### Consent to participate

Informed consent was obtained from all subjects involved in the study.

## Results

### STAG3 expression was up-regulated in CRC with STAG3 m6A methylation

**Figure 1:**
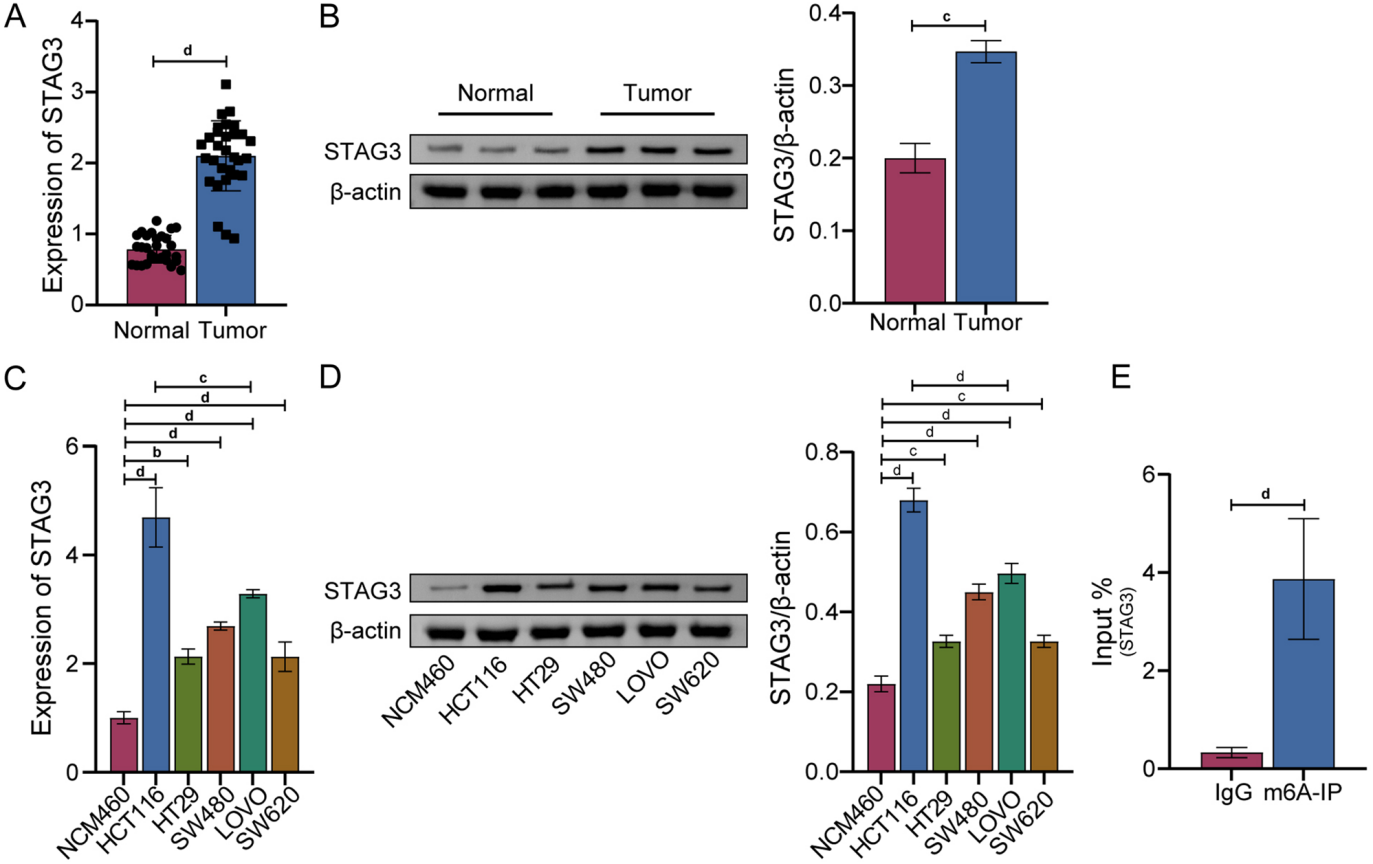
STAG3 expression was up-regulated in CRC with STAG3 m6A methylation. (**A,B**) The levels of STAG3 mRNA and protein expression in the CRC and adjacent normal tissues were evaluated using qRT-PCR and WB (n = 30). (**C,D**) The levels of STAG3 mRNA and protein expression level in the human normal colon cell line NCM460 and five popular CRC cell lines (HCT116, HT29, SW480, LOVO, SW620) were determined by WB. (**E**) Me-RIP was used to verify whether m6A methylation modification of STAG3 occurred in HCT116 cells. Superscript b: *P* < 0.01, superscript c: *P* < 0.001, superscript d: *P* < 0.0001.

We first used qRT-PCR to determine STAG3 expression in CRC tumors and the adjacent normal tissues. The outcomes revealed that the mRNA expression level of STAG3 in the Tumor group was much greater than it was in the Normal group (Fig. 1A). We further verified that the expression of STAG3 in CRC tissues was up-regulated at the protein level by WB (Fig. 1B). Furthermore, we also detected STAG3 expression at the cell level. The mRNA and protein expression of STAG3 in CRC cell lines was higher than that in NCM460 (Fig. 1C, D), which was consistent with the above results. Likewise, the expression of STAG3 in HCT116 was the highest. The Me-RIP experiment confirmed that m6A methylation modification occurred in STAG3 (Fig. 1E). The above results indicated that STAG3 was up-regulated in CRC and accompanied by STAG3 m6A methylation.

### METTL3 mediated m6A methylation of STAG3

**Figure 2:**
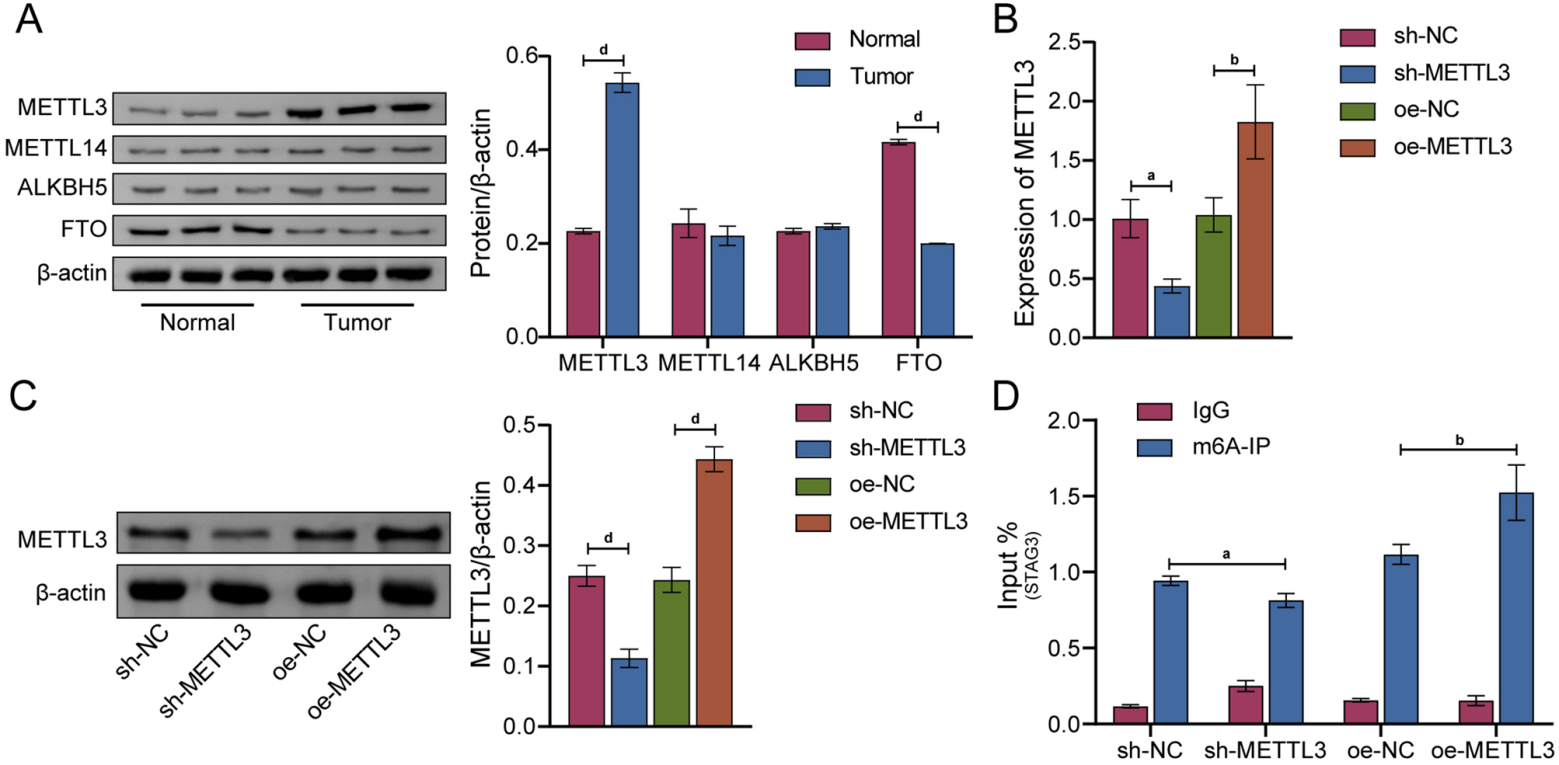
METTL3 mediated m6A methylation of STAG3. (**A**) In CRC and adjacent normal tissues, the protein expression levels of METTL3, METTL14, ALKBH5, and FTO were determined by WB. (**B,C**) The mRNA and protein expression levels of METTL3 in HCT116 cells were measured using qRT-PCR and WB. (**D**) The STAG3 m6A modification level in HCT116 cells was evaluated by Me-RIP. *P < 0.05 vs. normal, ^&^P < 0.05 vs. sh-NC, ^#^P < 0.05 vs. oe-NC. Superscript a: *P* < 0.05, superscript b: *P* < 0.01, superscript d: *P* < 0.0001.

The mechanism of m6A methylation modification of STAG3 in CRC was further explored. First, WB experiments was used to screen genes that regulate m6A methylation modification of STAG3. The analysis indicated that, in comparison to the Normal group, the Tumor group's expression of METTL3 was considerably up-regulated, that of FTO was down-regulated, and those of METTL14 and ALKBH5 were not significantly affected (Fig. 2A). The results of Fig. 2B, C showed that we successfully constructed METTL3 knockdown and METTL3 overexpression cell models. The m6A modification level of STAG3 was identified by Me-RIP assay. The outcomes showed that while the knockdown of METTL3 decreased the m6A level of STAG3, its overexpression led to the opposite results (Fig. 2D). The above findings demonstrated that METTL3 mediated m6A methylation modification of STAG3.

### METTL3 affected proliferation, migration, and apoptosis of CRC cells by regulating STAG3

**Figure 3:**
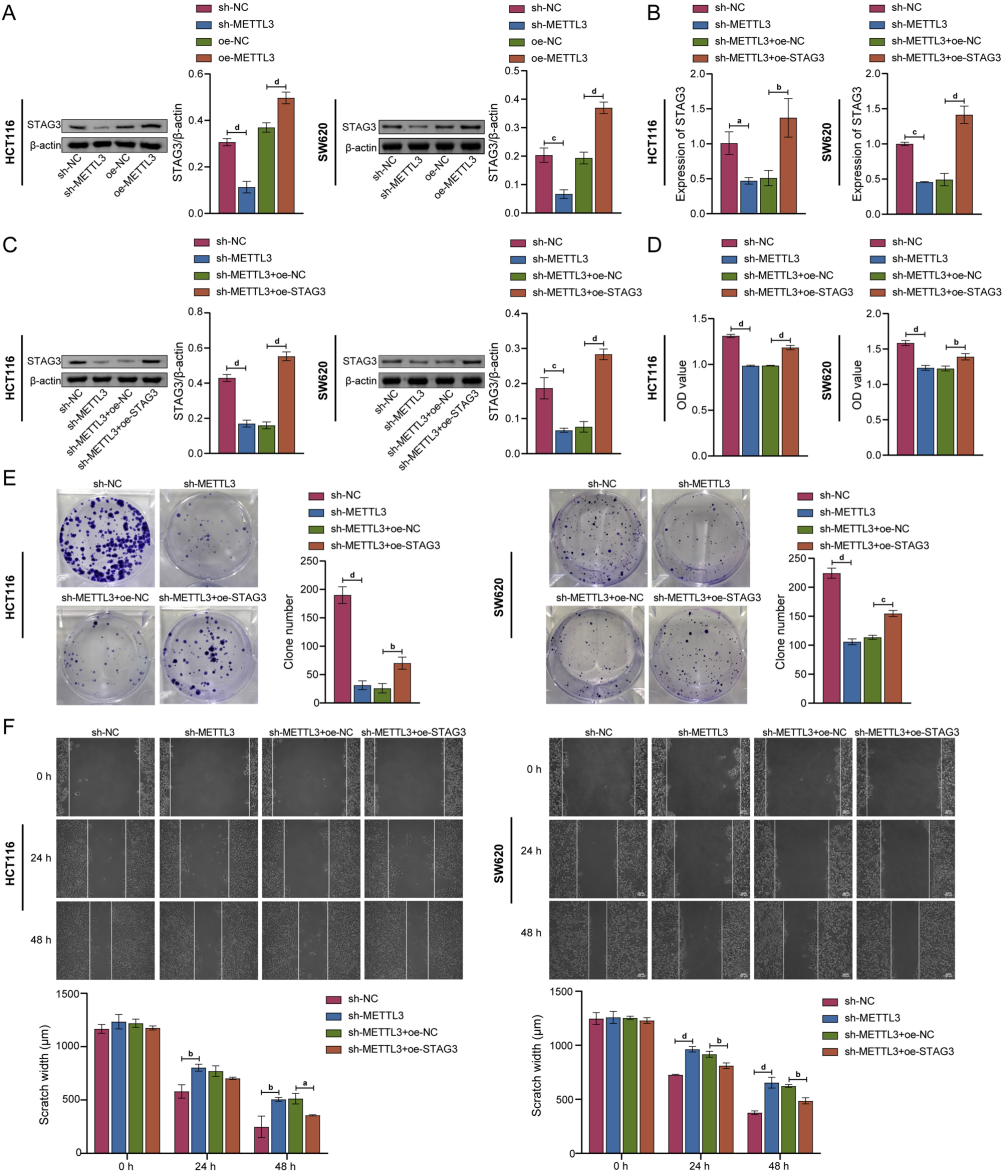

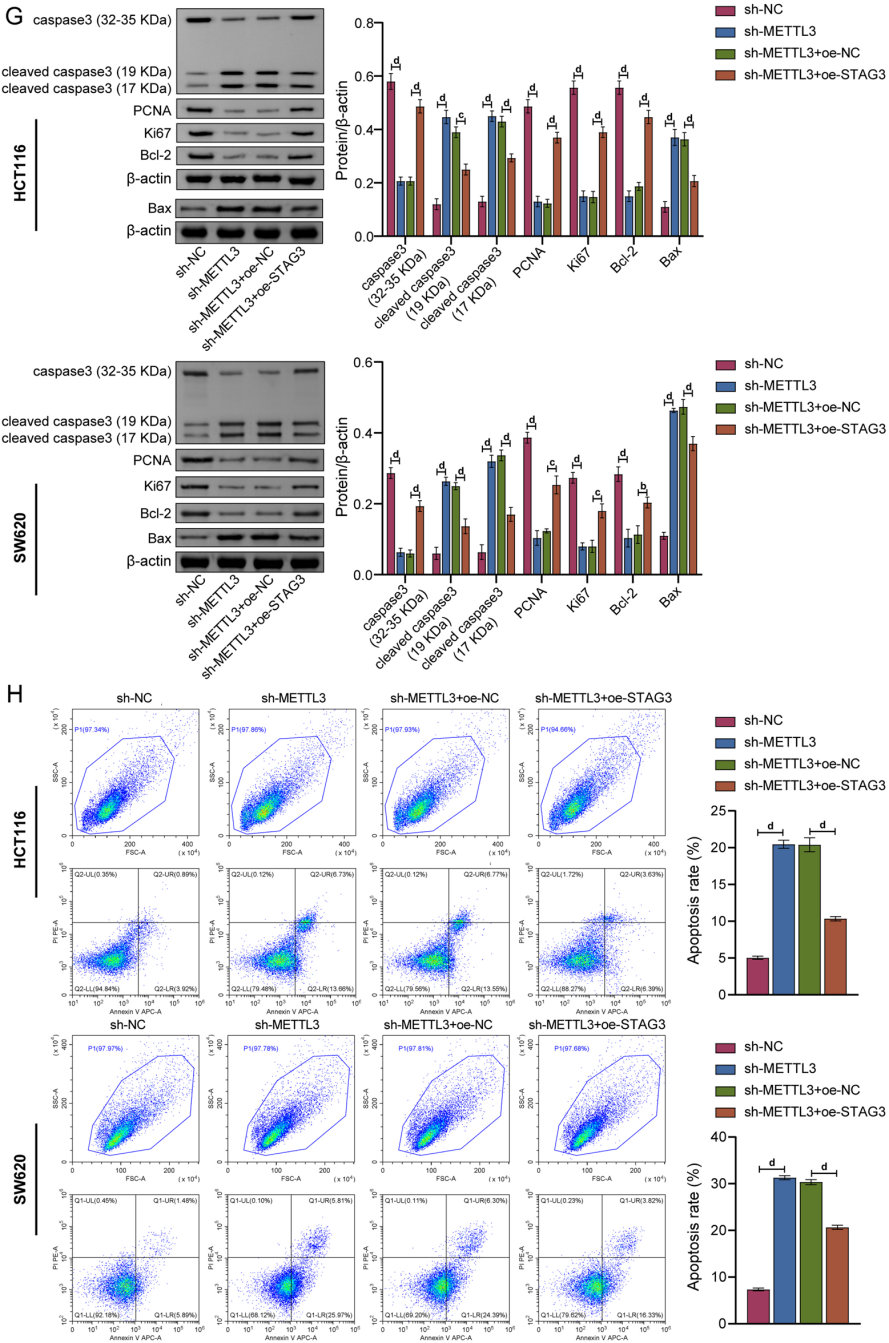
METTL3 affected proliferation, migration, and apoptosis of CRC cells by regulating STAG3. (**A**) WB was employed to assess the level of STAG3 protein expression in HCT116 and SW620 cells. (**B,C**) The mRNA and protein expression levels of STAG3 in HCT116 and SW620 cells were evaluated using qRT-PCR and WB, respectively. (**D**) Cell proliferation was discovered using the clone formation assay. (**E**) To study cell proliferation, the CCK-8 test was employed. (**F**) To examine cell migration, wound healing experiments were performed. (**G**) The protein expression levels of caspase3, cleaved caspase3, PCNA, Ki67, Bax, and Bcl-2 in HCT116 and SW620 cells were determined by WB. (**H**) Apoptotic cells were identified by flow cytometry. Superscript a: *P* < 0.05, superscript b: *P* < 0.01, superscript c: *P* < 0.001, superscript d: *P* < 0.0001.

Based on the aforementioned findings, HCT116 and SW620 cells were selected to further study the effects of METTL3 on the proliferation, migration and apoptosis of CRC cells and its regulatory mechanism. Therefore, METTL3 knockdown and STAG3 overexpression cell models were constructed. As seen in Fig. 3A, knockdown of METTL3 led to a drop in STAG3 protein expression levels, whereas overexpression of it had the opposite effect. This indicated that the expression of STAG3 was regulated by METTL3. Moreover, overexpression of STAG3 eliminated the effect on the mRNA and protein expression levels of STAG3 (Fig. 3B, C). CCK-8, colony formation, and wound healing data demonstrated that the knockdown of METTL3 inhibited cell proliferation, colony formation, and migration. However, these were reversed by overexpression of STAG3 (Fig. 3D–F). In the same vein, the effects of METTL3 on both the expression of proliferation-related proteins and apoptosis-related proteins were also detected by WB. As a result of METTL3 knockdown, the expression of caspase3 (32–35 KDa), PCNA, Ki67, and Bcl-2 were decreased. On the contrary, the expression of cleaved caspase3 (19 KDa), cleaved caspase3 (17 KDa), and Bax were increased. Whereas overexpression of STAG3 eliminated the effects of knockdown of METTL3 on these proteins (Fig. 3G). Results from flow cytometry demonstrated that knockdown of METTL3 promoted cell apoptosis, which was reversed by overexpression of STAG3 (Fig. 3H). According to the findings above, METTL3 affected the proliferation, migration, and apoptosis of CRC cells by regulating STAG3.

### IGF2BP2 was involved in the regulation of STAG3 expression by METTL3

**Figure 4:**
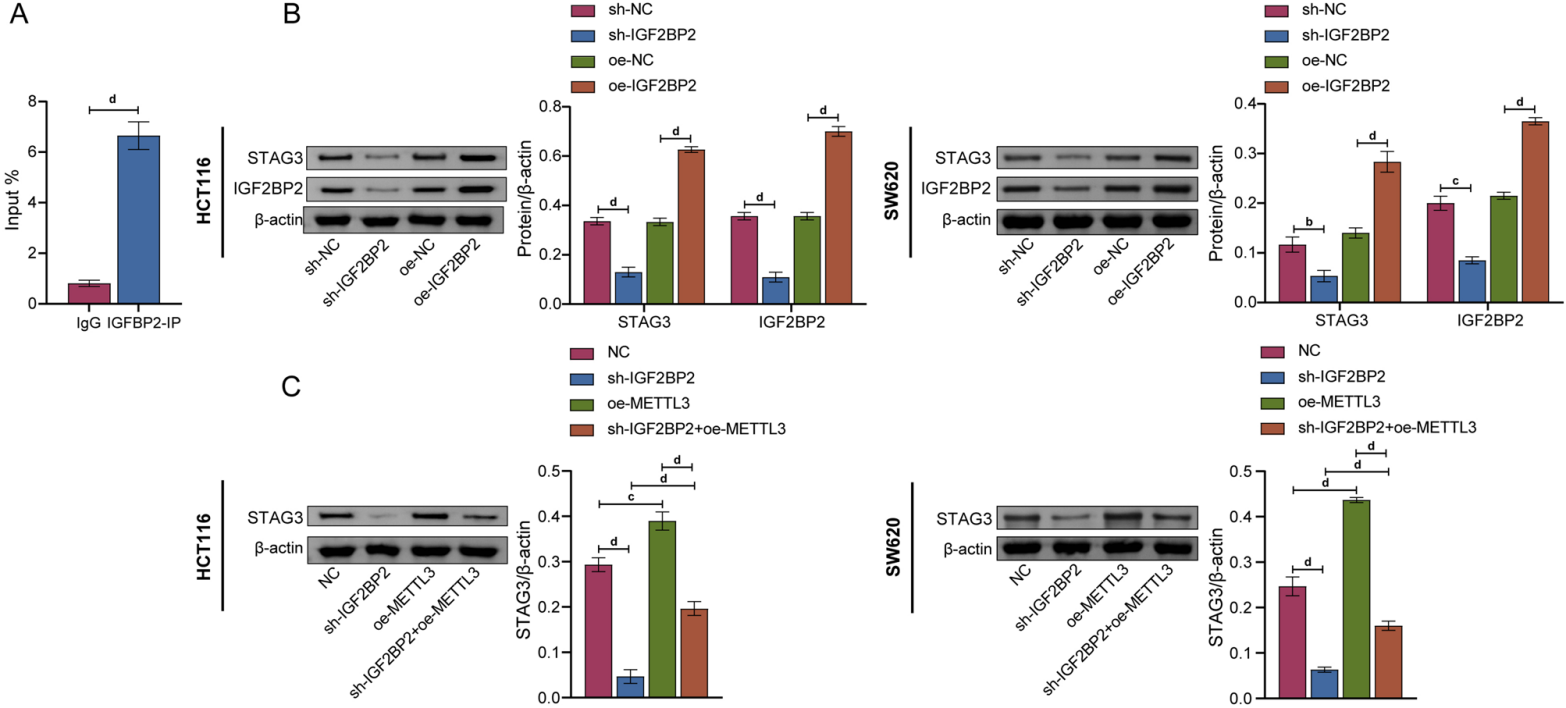
IGF2BP2 was involved in the regulation of STAG3 expression by METTL3. (**A**) The interaction between STAG3 mRNA and IGF2BP2 protein was discovered using the RIP assay. (**B**) WB was employed to assess the levels of STAG3 and IGF2BP2 protein expression in HCT116 and SW620 cells. (**C**) WB was employed to assess the level of STAG3 protein expression in HCT116 and SW620 cells. Superscript b: *P* < 0.01, superscript c: *P* < 0.001, superscript d: *P* < 0.0001.

Using catRAPID online prediction, we found that IGF2BP2, YTH domain-containing 2 (YTHDC2) and YTH domain family protein 3 (YTHDF3) may act on STAG3. This was further verified by RIP experiment. As shown in Fig. 4A, STAG3 mRNA interacted with IGF2BP2 protein, but, not with YTHDC2 and YTHDF3 (Figure S1). Likewise, WB results showed that after knockdown of IGF2BP2, the protein expression level of STAG3 declined. But, the reverse of this finding is observed after overexpression of IGF2BP2 (Fig. 4B). In addition, after overexpression of METTL3, the protein expression level of STAG3 was increased. Further knocking down IGF2BP2 reversed the effect of overexpression of METTL3. (Fig. 4C). These findings illustrated that IGF2BP2 was involved in the regulation of STAG3 expression by METTL3.

### IGF2BP2 affected proliferation, migration, and apoptosis of CRC cells by regulating STAG3

**Figure 5:**
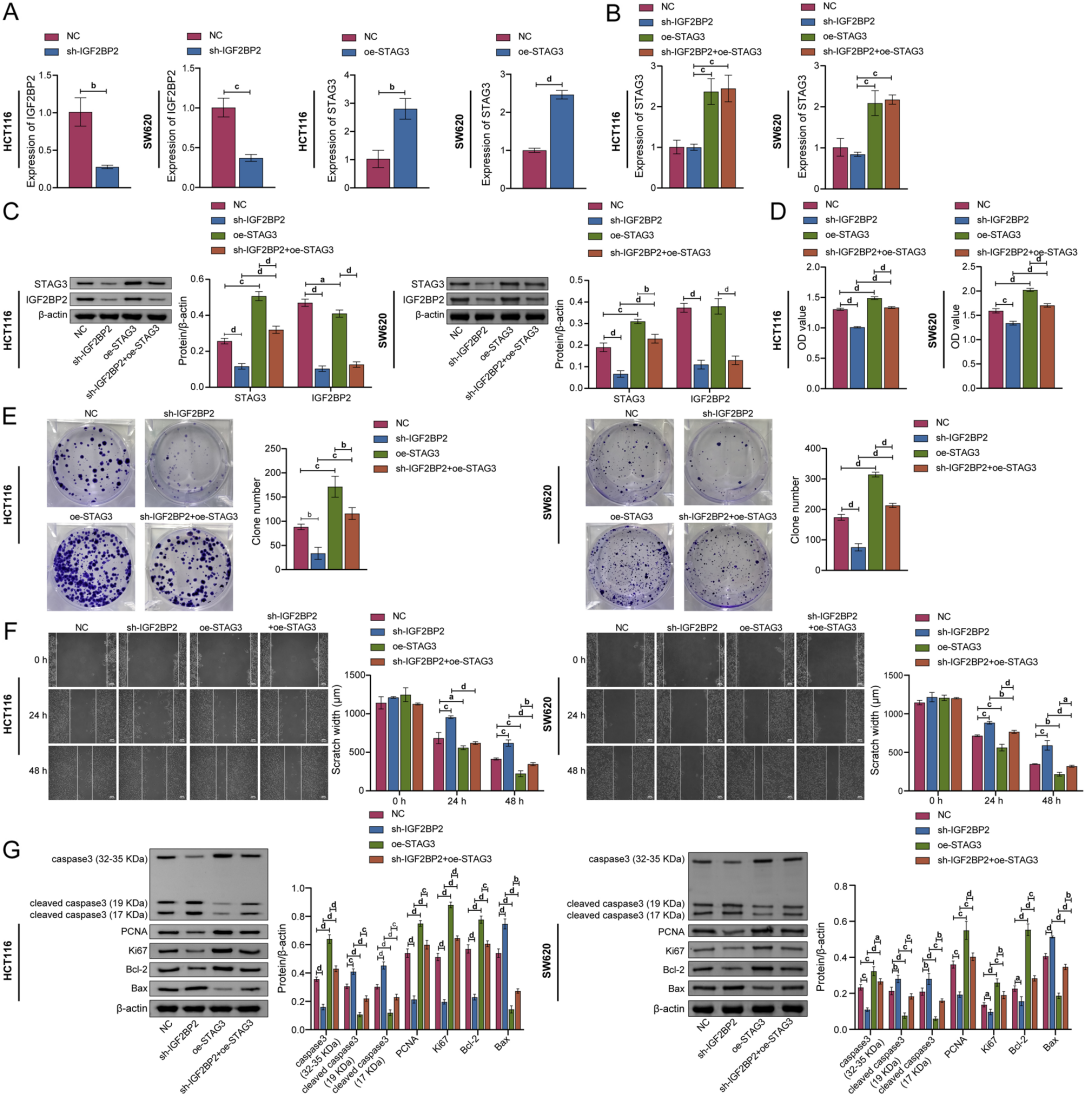
IGF2BP2 affected proliferation, migration, and apoptosis of CRC cells by regulating STAG3 translation. (**A**) The mRNA levels of IGF2BP2 and STAG3 in HCT116 and SW620 cells were analyzed utilizing qRT-PCR. (**B,C**) The mRNA and protein expression levels of STAG3 in HCT116 and SW620 cells were analyzed utilizing qRT-PCR and WB. (**D**) To study cell proliferation, the CCK-8 test was employed. (**E**) Cell proliferation was discovered using the clone formation experiment. (**F**) To examine cell migration, the wound healing test was utilized. (**G**) WB was adapted to identify the protein expression levels of caspase3, cleaved caspase3, PCNA, Ki67, Bax, and Bcl-2 in HCT116 and SW620 cells. (G) Apoptotic cells were identified by flow cytometry. Superscript a: *P* < 0.05, superscript b: *P* < 0.01, superscript c: *P* < 0.001, superscript d: *P* < 0.0001.

To further study the role of IGF2BP2 in the proliferation, migration, and apoptosis of CRC cells by regulating STAG3, we constructed IGF2BP2 knockdown and STAG3 overexpression cell models (Fig. 5A). As seen in Fig. 5B, C, the mRNA expression level of STAG3 was not significantly changed when IGF2BP2 was knocked down. Further overexpression of STAG3 led to a considerable increase in STAG3's mRNA expression level. WB was used to further identify the impact of IGF2BP2 knockdown on the level of STAG3 protein expression. The findings demonstrated that the protein expression level of STAG3 decreased after knockdown of IGF2BP2, and increased after overexpression of STAG3 (Fig. 5D). The abilities of cell proliferation and migration were all decreased by knockdown of IGF2BP2 in CCK-8, colony formation, and wound healing experiments. In contrast, the overexpression of STAG3 reversed the levels of these indices (Fig. 5E, F). Furthermore, WB was used to investigate the expression of the apoptosis-related proteins as well as the proliferation-related proteins. The results demonstrated that cleaved caspase3 (17 KDa), cleaved caspase3 (15 KDa), and Bax expressions were elevated following knockdown of IGF2BP2. On the contrary, caspase3 (32–35 KDa), PCNA, Ki67, and Bcl-2 expressions were reduced. Overexpression of STAG3 eliminated the effect of IGF2BP2 knockdown on the expression of these proteins (Fig. 5G). The above results indicated that IGF2BP2 regulated STAG3 and affected CRC cell proliferation, migration, and apoptosis.

### METTL3 affected CRC progression by regulating STAG3 in vivo

**Figure 6:**
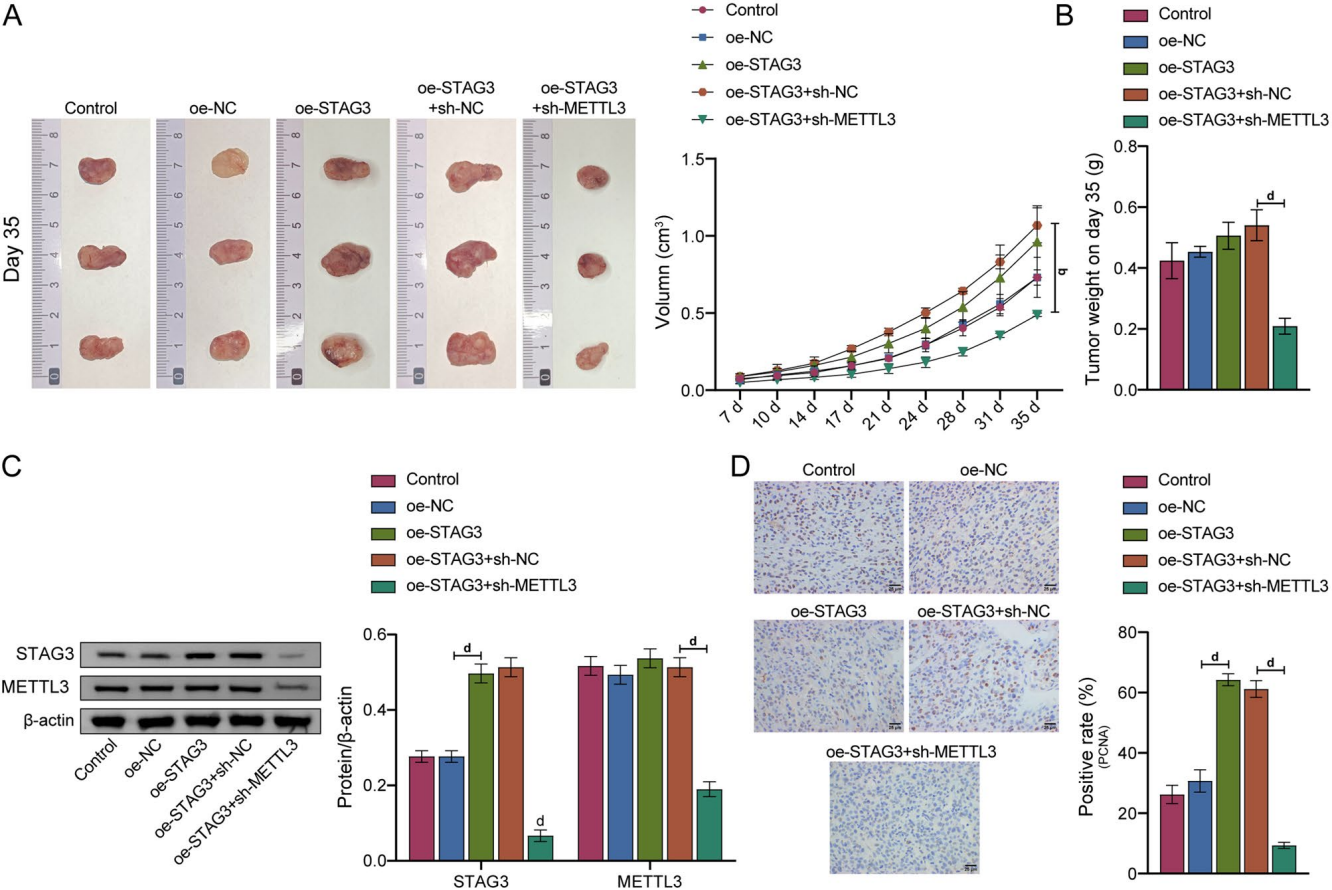
METTL3/IGF2BP2 signaling axis affected CRC progression by regulating STAG3 in vivo*.* (**A**) Tumor images and growth curves. (**B**) Tumor weight. (**C**) In CRC nude mice, WB was adapted to identify the levels of STAG3 and METTL3 protein expression. (**D**) To identify PCNA expression in CRC nude mice, IHC was employed. ^d^*P* < 0.0001.

Subcutaneous tumorigenesis experiment was conducted in nude mice in order to verify that METTL3 affects CRC progression by regulating STAG3 in vivo. The volume and weight of tumors did not change significantly following the overexpression of STAG3. But, a subsequent knockdown of METTL3 resulted in a considerable decrease in both parameters, as seen in Fig. 6A, B. In nude mice, STAG3 protein expression level increased following overexpression of the gene, but, METTL3 did not change appreciably, according to WB data. Following further knockdown of METTL3, the expression levels of STAG3 and METTL3 decreased (Fig. 6C). Similarly, IHC was used to identify the expression of PCNA in nude mice. The findings demonstrated that overexpression of STAG3 caused PCNA expression to increase, while further knockdown of METTL3 caused PCNA expression to decline (Fig. 6D). The above results indicated that METTL3 affected CRC progression by regulating STAG3 in vivo*.*

## Discussion

CRC is the most common gastrointestinal malignancy that affects humans. Also, it is ranked among the malignancies with the highest morbidity and death^1^. Poor prognosis for CRC is linked with increased STAG3 expression^5^. But, the regulatory mechanism of STAG3 in CRC remains to be studied. Our findings demonstrated that STAG3 was up-regulated in CRC and influences the proliferation, migration, and apoptosis of CRC cells via the molecular process of m6A modification. In addition, METTL3 and IGF2BP2 regulated STAG3 expression. Collectively, our study revealed that STAG3 affected the progression of CRC through the METTL3/IGF2BP2/STAG3 axis. This could serve as a novel predictive biomarker or potent treatment target for CRC.

STAG3, the main subunit of the meiosis-specific adhesion protein complex, controls sister chromatid adhesion^4^. STAG3 promotes chromosome separation and sister chromatid formation^23^ which ensures correct DNA repair and chromosome separation^24^. When dysregulated, STAG3 gene can block the aggregation of sister chromatids, and cause chromosome instability. This effect may promote tumor development^25^. Zhao et al. conducted an investigation into the expression of STAG3 in hepatocellular carcinoma (HCC) tissue using IHC and qRT-PCR. They observed a downregulation of STAG3 expression in HCC, and further found that lower STAG3 expression was associated with advanced clinical pathological features and poor prognosis^26^. In contrast, Sasaki et al. evaluated the expression of STAG3 mRNA and protein in CRC tissue through qRT-PCR and immunohistochemistry, discovering a high expression of STAG3 in CRC tissue. They also noted a correlation between elevated STAG3 expression and poor prognosis in CRC patients^5^. These findings align with our study in which we assessed STAG3 expression in CRC tissue and cell lines using qRT-PCR and WB analysis, detecting a high expression of STAG3. This suggested that STAG3 might exhibit heterogeneity and undergo complex transcriptional regulation in different tumor types. However, our results differed from the analysis of the TCGA COAD cohort, comprised of 41 adjacent normal tissues and 286 CRC tissues, as it unveiled a downregulated expression of STAG3 in CRC. We hypothesized that this discrepancy might stem from tissue-specific differences between samples or the influence of distinct research methods and experimental conditions.

m6A, an epigenetic regulation mechanism, is an emerging research frontier in tumor biology^27,28^. Cui et al. demonstrated that m6A methylation modification is critical for the self-renewal and carcinogenesis of glioblastoma stem cells^29^. Similarly, Zhang et al. found that ZNF217 promoted the phenotype of breast cancer stem cells by inhibiting the m6A modification of pluripotent factors in hypoxic breast cancer cells^30^. In CRC, m6A modification was found to be closely related to the activation of glycolysis pathway^31^. However, whether STAG3 expression in CRC is regulated by m6A modification and its related enzymes remains to be studied. In this investigation, Me-RIP test confirmed that STAG3 had m6A methylation modification. According to these findings, STAG3 expression is up-regulated in CRC along with STAG3 m6A methylation.

METTL3 is the most critical enzyme in the process of m6A methylation. There are few studies on METTL3 in CRC. The majority of these studies have concluded that METTL3 is highly expressed at various levels in CRC tissues and CRC cell lines. Also, this high expression is strongly associated with the dismal prognosis of CRC patients^17,32,33^. For example, Li et al. found that METTL3 promoted the progression of CRC by enhancing the stability of SOX2 mRNA in a m6A-IGF2BP2-dependent manner^17^. According to Chen et al., METTL3 might be a therapeutic target for CRC since it activates m6A-GLUT1-mTORC1 and promotes CRC development^32^. Likewise, Peng et al. found that up-regulation of METTL3 promoted CRC metastasis through miR-1246/SPRED2 targeting MAPK signaling pathway^33^. Our analysis found that METTL3 was highly expressed in CRC tissues, which is in line with the findings of the above studies. Additionally, we discovered that knockdown of METTL3 reduced the m6A modification level and protein expression level of STAG3, while overexpression of METTL3 had the opposite effect. These indicated that METTL3 controlled the expression of STAG3 and facilitated the m6A methylation modification of STAG3. Using in vivo experiments, we were able to determine that knockdown of METTL3 decreased cell proliferation and migration while promoting cell apoptosis. Furthermore, we also demonstrated that METTL3 affected CRC progression by regulating STAG3 in vitro. According to our findings, METTL3 modulated STAG3 both in vivo and in vitro, which had an impact on the biological function of CRC.

Preliminary research suggested that m6A readers play a critical role in cancer development and prognosis^34^. For example, in CRC patients, low expression of YTHDC2 is associated with poor prognosis^35^, while upregulation of YTHDF3 inhibits CRC progression^36^. High expression of IGF2BP2 also plays a role in CRC^20,21^. To investigate the interactions between these readers and STAG3, we first conducted RIP experiments, but the results showed that YTHDC2 and YTHDF3 did not interact with STAG3. Therefore, IGF2BP2 was selected for further study. It should be noted that, we did not compare the expression levels of these readers in CRC, which is a limitation of the study. IGF2BP2 is considered to be a key "reader" of m6A modification. Also, it is implicated in the development of numerous malignant tumors by post-transcriptional regulation of the stability and translation of its key target mRNA^18,19^. The role of IGF2BP2 as a tumor promoter in the advancement of cancer is supported by the experimental data gathered so far. Furthermore, its overexpression and amplification are related to cancer development and indicate a poor prognosis in many malignancies^37^. However, the fundamental mechanism through which IGF2BP2 promotes cancer cell survival, proliferation, and migration is yet unknown^38,39^. According to earlier research, IGF2BP2 is a crucial tumor-promoting factor in the development of CRC^20^. Our research revealed that IGF2BP2 was implicated in the control of STAG3 by METTL3, and regulation of STAG3 protein expression. It is worth noting that, the experiment found that IGF2BP2 knockdown led to a decrease in STAG3 protein level although STAG3 mRNA level only showed a non-significant change. This might be attributed to the stronger effect of IGF2BP2 as an RNA-binding protein compared to its effect as an m6A reader^40^, meaning that IGF2BP2’s influence on STAG3 protein translation is greater than its impact on mRNA transcription. Further details of the influence of IGF2BP2 on the biological processes of CRC cells was also examined. It was discovered that knockdown of IGF2BP2 reduced cell proliferation and migration while promoting apoptosis. But these impacts were reversed when STAG3 was overexpressed. These findings showed that IGF2BP2 regulated STAG3 translation to influence CRC cell proliferation, migration, and apoptosis.

In this work, we validated the high expression of STAG3 in CRC and elucidated another detailed mechanism involving STAG3 in CRC. Specifically, we demonstrated that STAG3 affects CRC progression through the m6A modification-dependent mechanism mediated by the METTL3/IGF2BP2 axis. This represents the novelty and innovation of our study. Nonetheless, there are few studies on STAG3 in CRC, and its exact regulatory mechanism still needs to be analyzed in a sufficient number of CRC clinical and animal samples. Overall, our results highlighted the potential of STAG3 as a novel predictive biomarker or effective therapeutic target. It expands the repertoire of potential targets for targeted therapy in CRC patients.

### Supplementary Information

Supplementary Figure S1.

Supplementary Information.

## Data Availability

The data used to support the findings of this study are available from the corresponding author upon request.
