# Supplementary material for: METTL3/IGF2BP2 axis affects the progression of colorectal cancer by regulating m6A modification of STAG3
Source: Sci Rep. 2023 Oct 12;13:17292. doi: 10.1038/s41598-023-44379-x (PMC10570365; doi:10.1038/s41598-023-44379-x)
Supplement: Supplementary file 1 — Supplementary Figure S1. [file 41598_2023_44379_MOESM1_ESM.docx]

**
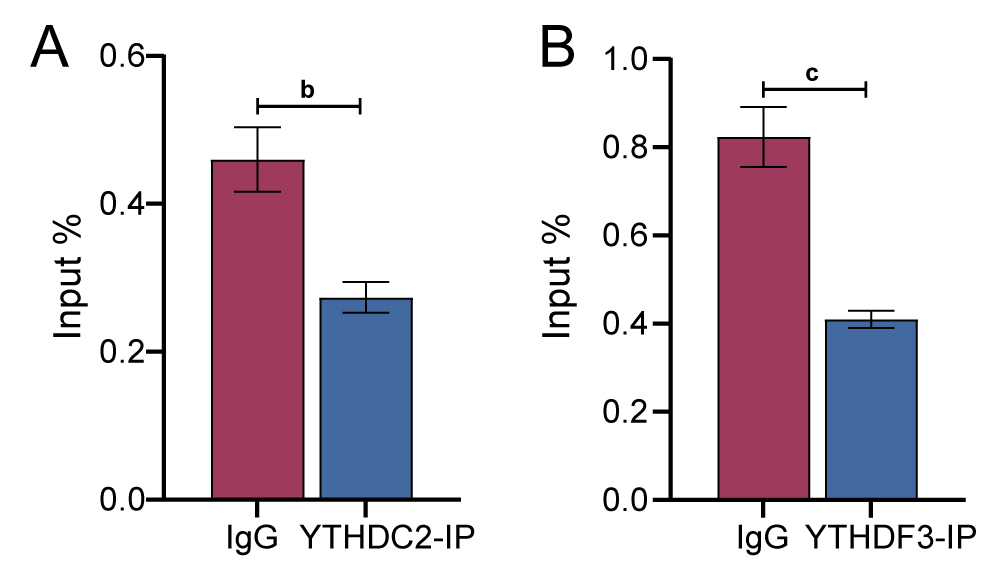
**

**Supplementary Figure S1** The interaction between STAG3 mRNA and m6A “reader” protein (YTHDC2 and YTHDF3) was discovered using the RIP assay. ^b^*P*<0.01, ^c^*P*<0.001.
