# Supplementary material for: METTL3/IGF2BP2 axis affects the progression of colorectal cancer by regulating m6A modification of STAG3
Source: Sci Rep. 2023 Oct 12;13:17292. doi: 10.1038/s41598-023-44379-x (PMC10570365; doi:10.1038/s41598-023-44379-x)
Supplement: Supplementary file 2 — Supplementary Information. [file 41598_2023_44379_MOESM2_ESM.pdf]

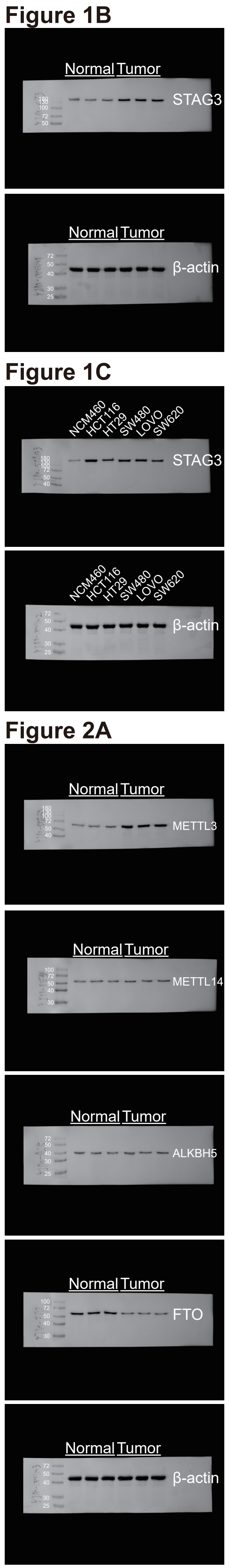

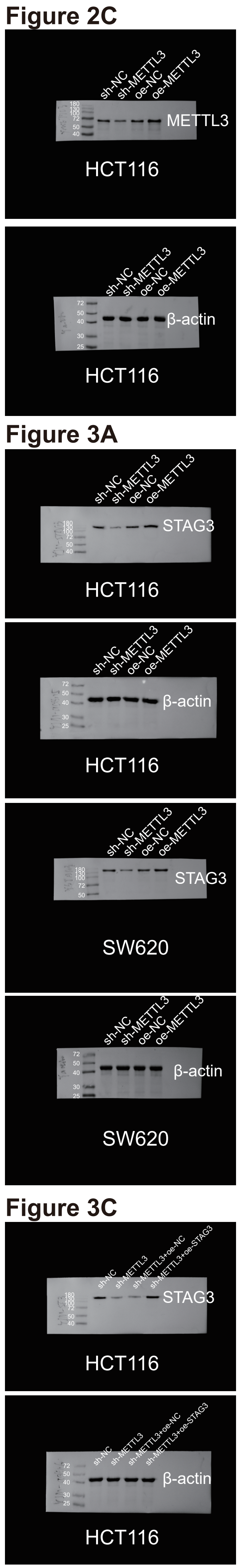

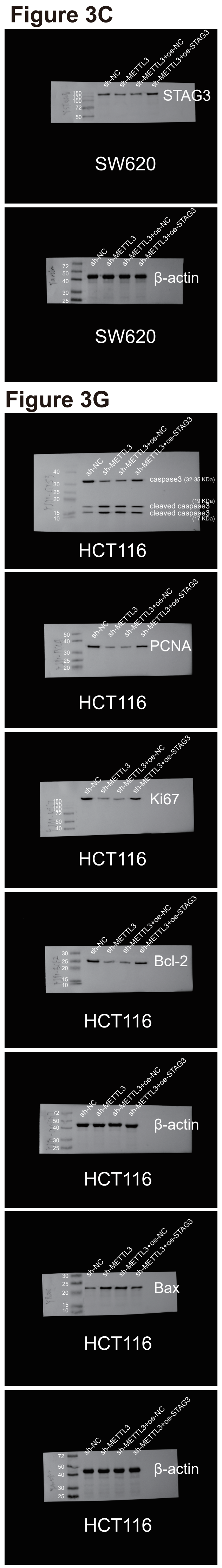

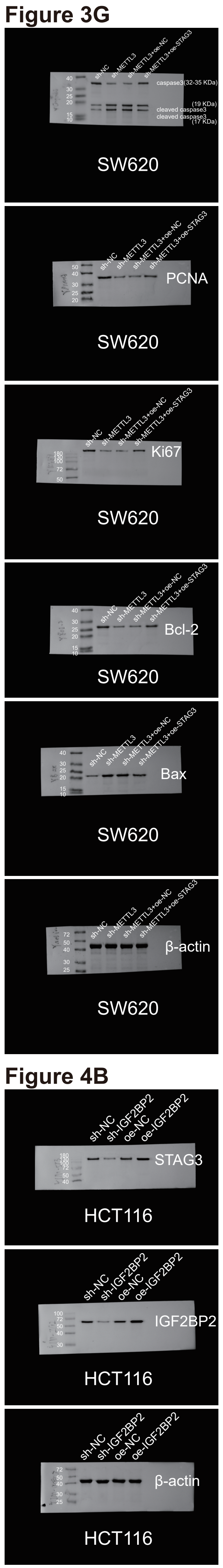

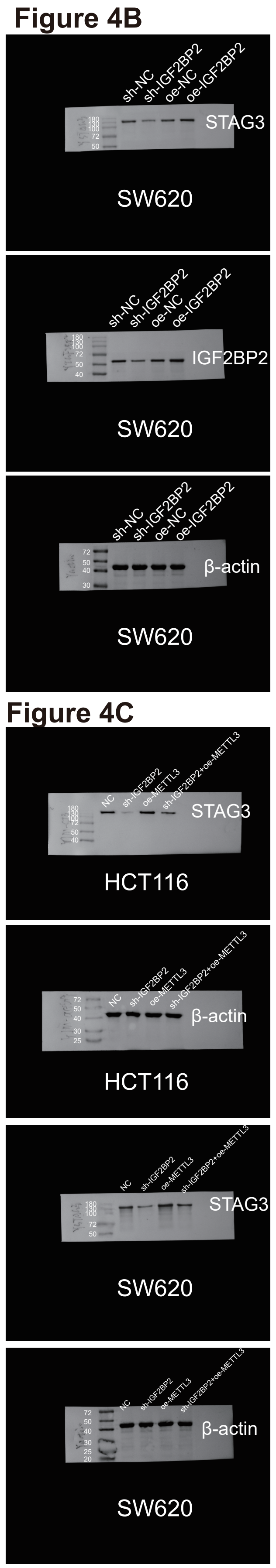

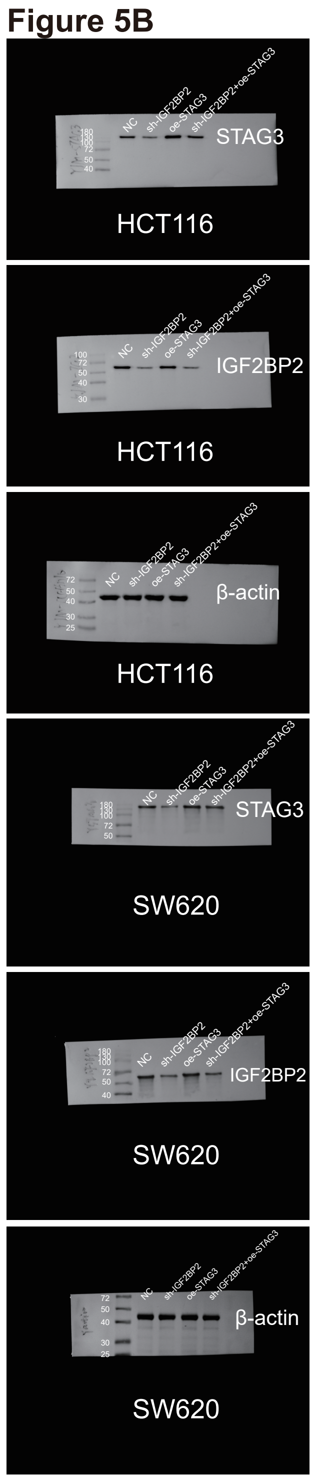

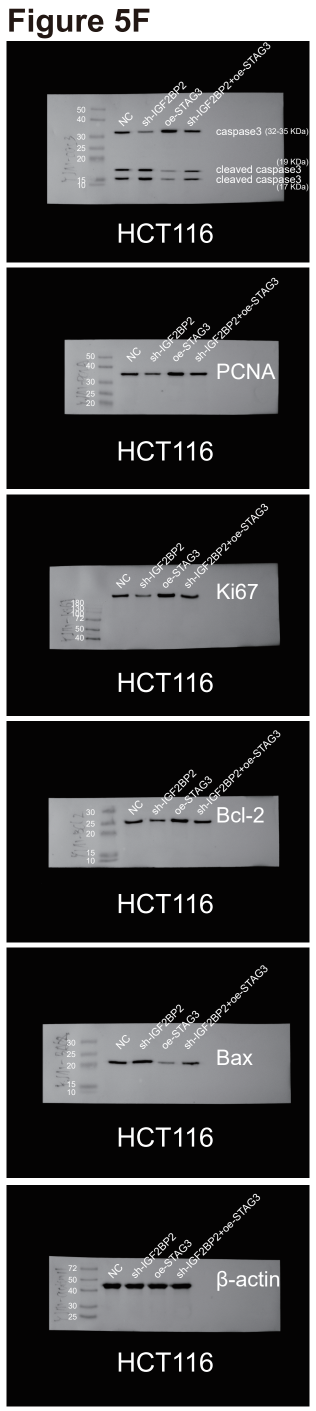

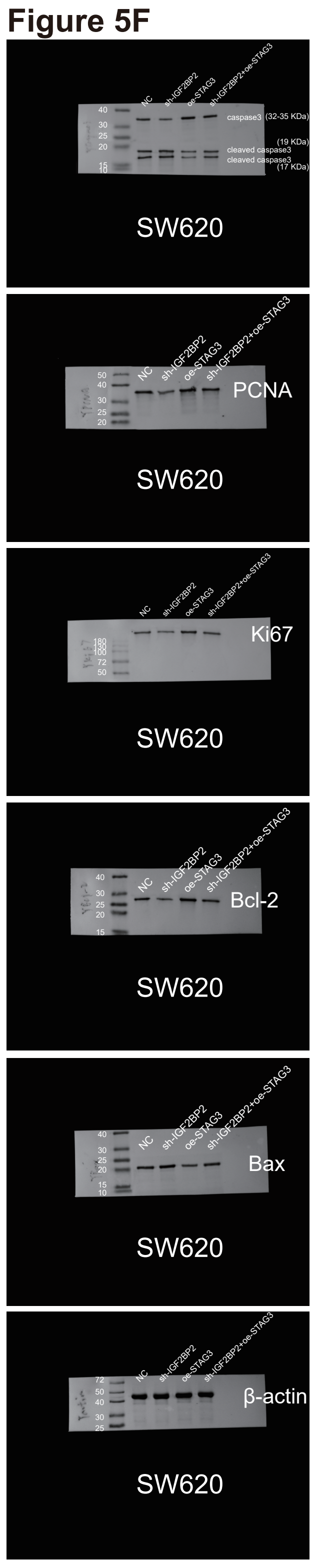

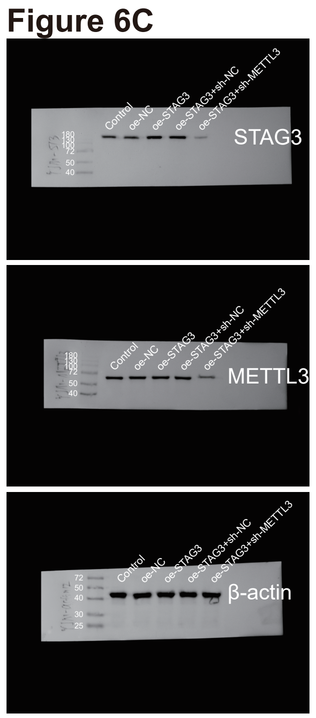

Supplementary File S1 Full-length blots.
